# Experimental Researches Applied to Physiology and Pathology

**Published:** 1852-10

**Authors:** E. Brown-Séquard

**Affiliations:** Paris


MEDICAL EXAMINER,

AND

RECORD OF MEDICAL SCIENCE.

NEW SERIES.—NO. XCIV.—OCTOBER, 1852.

### ORIGINAL COMMUNICATIONS.

### *Experimental Researches applied to Physiology and Pathology.*

By E. BROWN-SÉQUARD, M. D., of Paris.

(Continued.)

### XVII.—ON THE INFLUENCE OF THE TEMPERATURE OF A WARM-BLOODED ANIMAL UPON THE DURATION OF ITS LIFE WHEN IT IS ASPHYXIATED.

One of the most positive facts in physiology is that every animal needs oxygen in order to live. But if there is a complete uniformity as to the necessity of oxygen for all animals, there is also the greatest variety between the different species as to the quantity of that gas which is necessary for the maintenance of life. Very probably a great part of the physiological differences between different animals, comes from the difference in the quantity of oxygen absorbed by their blood in a given time. The most important cause of the differences existing between cold and warm-blooded animals, and between young and adult animals, is to be found in the differences in the quantity of oxygen they absorb.

It is very remarkable that one of the principal laws relative to the anatomical differences existing between the different species of animals, appears to be also a law regulating their physiological differences. In an anatomical point of view, a mammal, at the different periods of its development, presents alternately the forms of many different beings. In a physiological point of view, a corresponding transformation occurs for the mammals. They exhibit alternately the same phenomena of life exhibited by many other different animals. For instance, before its birth, the mode of breathing of a mammal is the same as that of fishes. The oxygen absorbed by fishes exists in a liquid; the oxygen absorbed by the mammal fœtus exists also in a liquid, which is the blood of the mother. In fishes and also in mammal fœtuses, oxygen has to pass through many membranes: in fishes, through the mucous membrane of the bronchiæ and the membrane of the capillary vessels; in the mammal fœtuses, through the membranes of the capillary vessels of the maternal and the fœtal placentas, and their mucous coverings. After birth young mammals have an insufficient power of breathing, and in this they are like the reptilia. They are unable to absorb a sufficient quantity of oxygen to resist the influence of cold; and their power of retaining life when deprived of oxygen, is comparable to that of the reptilia.

This fact has been well established by the experiments of Buffon, Boyle, Ens, Roose, Haller, Fontana, Legallois, and more particularly W. F. Edwards. Nevertheless, these eminent experimenters have left many important questions without solution, some of which I will examine here.

In order to study the influence of temperature on the duration of life in asphyxiated animals, W. F. Edwards dipped into water, at different degrees of temperature, many animals of different ages. Unhappily he did not take notice of the temperature of the animals on which he experimented; and we will show that this circumstance is very important, because the degree of that temperature at the instant when asphyxia begins, has a considerable influence on the duration of life. Consequently, to discover with exactitude the influence of the temperature of a medium, on animals deprived of breathing, it was necessary to operate on animals at the same temperature. This has been done neither by Edwards nor by any other physiologist.

*A priori* it is easy to acknowledge that the duration of life in animals asphyxiated can be influenced by four capital circumstances: 1st. The degree of the temperature of the animals; 2d. The degree of the temperature of the medium; 3d. The age of the animals; 4th. Their species.

Consequently four series of experiments were to be performed. I have made them, and I propose to give here some of the results I have obtained. The remaining shall be detailed in a special paper.

### I. *Influence of the Temperature of young warm-blooded animals on the length of their resistance to Asphyxia.*

*Experiment* 1. Nine rabbits of the same brood and aged about two days, were dipped into water at 25° Cent. (77° Fahr.) Two of these animals, No. 1 and No. 2, had the temperature which they generally have when they are in their nest covered by their mother. The others had been cooled by having been exposed to the action of an atmosphere at 10° Cent. (50° Fahr.) No. 3 and No. 4 had been exposed for a quarter of an hour; No. 5 and No. 6 for three quarters of an hour; and No. 7, No. 8 and No. 9 for an hour and a half. The results are represented in the following table:

TABLE I.*

| Nos. | Temperature of Animals. | | Duration of Life. | Mean Duration of Life. |
|---|---|---|---|---|
| 1, | 35° to 36° Cent. | (95 to 97° Fahr.) | 10′ | 12′ |
| 2, | ditto | ditto | 14′ | |
| 3, | 29° to 30° Cent. | (84° to 86° Fahr. | 16′ | 18′½ |
| 4, | ditto | ditto | 21′ | |
| 5, | 23° to 24° Cent. | (74° to 75° Fahr.) | 20′ | 23′ |
| 6, | ditto | ditto | | |
| 7, | 18° to 19° Cent. | (65° to 66° Fahr.) | 22′ | 28′ |
| 8, | ditto | ditto | 29′ | |
| 9, | ditto | ditto | 33′ | |

The one that lived shortest (10′,) was nearly at 36° Cent.

* The existence of reflex movements has been used in this experiment and in all the others as the proof of life.

(97° Fahr.;) the one that lived the longest (33′,) was nearly at 18° Cent. (65° Fahr.) The difference of temperature being 17° or 18° Cent. (about 32° Fahr.) the difference as to the duration of life was 23 minutes—that is, nearly two minutes for each diminution of three degrees Fahr.

The same experiment performed on many breeds of rabbits, has always given very nearly the same results.

These experiments prove that the temperature of young rabbits has a decided influence upon the duration of their life, when they are deprived of breathing. I have ascertained that the same thing takes place in cats, dogs, mice, and many species of birds.

*Experiment* 2. I dipped into water at the temperature of 25° Cent. (77° Fahr.) three bull-dogs.

TABLE II.

| Nos. | Temperature of Animals. | | | Duration of Life. |
|---|---|---|---|---|
| 1, | 38° Cent. (about | 101° | Fahr.) | 15′ |
| 2, | 30 " | 86 | " | 24′ |
| 3, | 22 " | 77 | " | 47′ |

Between No. 1 and No. 3 the difference of temperature was 16° Cent. (29° Fahr.;) the difference in the duration of life was thirty-two minutes—that is, two minutes for each diminution of one centigrade degree (nearly 2° Fahr.)

*Experiment* 3. I put a ligature around the trachea of four cur-dogs of the same brood, and aged three days.

TABLE III.

| Nos. | Temperature of Animals. | | | Duration of Life. |
|---|---|---|---|---|
| 1, | 37° Cent. (about | 99° | Fahr.) | 13′ |
| 2, | 28 " | 83 | " | 19′ |
| 3, | 24 " | 76 | " | 31′ |
| 4, | 19 " | 67 | " | 51′ |

So that between the two extremes the difference as the duration of life, was 38 for 18 Cent. (about 32° Fahr.)

Many other experiments on very young dogs gave very nearly the same results.

*Experiment* 4. On five cats, of the same brood, and aged two days, I put a ligature around the trachea, after having cooled three of them.

TABLE IV.

| Nos. | Temperature of the animals. | Duration of life. | Mean duration. |
|---|---|---|---|
| 1, | 36° Cents., (about 97° Fahr.) | 21′ | 25½′ |
| 2, | 36 " " 97 " | 30 | |
| 3, | 23 to 24 Cent., (74 to 76 Fahr.) | 47 | 48½ |
| 4, | 22 to 23 Cent., (72 to 75 Fahr.) | 50 | |
| 5, | 17 Cent., (about 63 Fahr.) | | 53 |

The temperature of the air air was then at 18¼° Cents., (65 Fahr.)

*Experiment* 5. On four mice, probably aged from three to eight days, I put a ligature around the trachea after having cooled three of them.

TABLE V.

| Nos. | Temperature of the animals. | Duration of life. |
|---|---|---|
| 1, | 34° Cents., (about 94° Fahr.) | 11′ |
| 2, | 27 " " 81 " | 14 |
| 3, | 22 " " 72 " | 10 |
| 4, | 18 " " 65 " | 14 |

I have obtained analogous results in experimenting on birds.

*Experiment* 6. On seven magpies, aged from four to eight days, I put a ligature around the trachea, and opened widely the thoraco-abdominal cavity.

TABLE IV.

| Nos. | Temperature of the animals. | Duration of life. | Mean duration. |
|---|---|---|---|
| 1, | 36° Cent., (about 97° Fahr.) | 20′ | 24, |
| 2. | 36 " " 97 " | 28 | |
| 3, | 32 " " 97 " | 26 | 32½ |
| 4, | 31½ " " 90 " | 39 | |
| 5, | 26 " " 79 " | 12 | 50 |
| 6, | 26 " " 79 " | 58 | |
| 7, | 22 " " 72 " | | 72 |
| 8, | 20 " " 68 " | 65 | 76 |
| 9, | 20 " " 68 " | 87 | |
| 10, | 18 " " 65 " | 82 | 92 |
| 11, | 10 " " 65 " | 103 | |

*Experiment* 7. On other magpies I made the same experiment, but without opening the thoraco-abdominal cavity, so that asphyxia was much more complete.

TABLE VII.

| Nos. | Temperature of the animals. | Duration of life. |
|---|---|---|
| 1, | 35° Cent., (95° Fahr.) | 8′ |
| 2, | 30 " 86 " | 15 |
| 3, | 24 " 76 " | 27 |
| 4, | 19 " 67 " | 39 |

I have obtained nearly the same results from experiments upon many sparrow-hawks, ravens and jays.

All these facts show how considerable is the influence of the temperature of certain young animals and birds on the duration of their life when they are asphyxiated.

II. *Differences in the length of the resistance to asphyxia according to the species of animals.*

In order to discover what is the influence of the species, I made the following experiment. I tied the trachea on twenty-two animals belonging to eleven species, all of which were aged about four or five days. Their temperature had been cooled and they were all at about 26° Cent., (79° Fahr.) The temperature of the air was 19° Cent. (67° Fahr.)

TABLE VIII.

| Nos. | Species. | Duration of life. | Mean duration of life |
|---|---|---|---|
| 1, | Rabbit | 18′ | 21′ |
| 2, | do. | 24 | |
| 3, | Guinea pig | 6 | 7½ |
| 4, | do. | 9 | |
| 5, | Mouse | 20 | 23½ |
| 6, | do. | 27 | |
| 7, | Dog | 25 | 32 |
| 8, | do. | 39 | |
| 9, | Cat | 31 | 33½ |
| 10, | do. | 36 | |
| 11, | Sparrow | 8 | 10 |
| 12, | do. | 12 | |
| 13, | Pigeon | 5 | 7 |
| 14, | do. | 9 | |
| 15, | Jay | 13 | 15 |
| 16, | do. | 17 | |
| 17, | Raven | 14 | 18½ |
| 18, | do. | 23 | |
| 19, | Sparrow Hawks | 16 | 20 |
| 20, | do. | 24 | |
| 21, | Mag-pie | 18 | 22½ |
| 22, | do. | 25 | |

Now if we compare birds to mammals, we see that generally mammals resist asphyxia more than birds. The two following tables show the difference:

TABLE IX.

Mammals.

| | |
|---|---|
| Guinea-pigs | 7½′ |
| Rabbits | 21 |
| Mice | 23½ |
| Dogs | 32 |
| Cats | 33½ |
| Average | 23½ |

TABLE X.

Birds.

| | |
|---|---|
| Pigeons | 7′ |
| Sparrows | 10 |
| Jays | 15 |
| Raven | 18½ |
| Sparrow Hawks | 20 |
| Mag-pies | 21½ |
| Average | 15¼ |

### III. *Influence of the temperature of adult warm-blooded animals on the duration of life when they are asphyxiated.*

I have discovered that the degree of the temperature of adult non-hybernating vertebrata has also a great influence on their power of resisting asphyxia. Dogs, cats, rabbits, guinea-pigs and birds, are subject to the same law when they are adult as when they are very young. The lower their temperature, the longer they live when they are asphyxiated. But although the existence of this law for adult vertebrata is beyond all doubt, it is sometimes very difficult to ascertain that existence. It is not easy to diminish the temperature of a non-hybernating adult mammal, or that of a bird, without exhausting the nervous and the muscular power of the animal, and also without producing a general and considerable perturbation. The action of a cold bath, for instance, so powerfully excites the vertebrata, that sometimes violent convulsions take place, and then death may occur in a short time, even before the animal has lost 10° cent. (18° Fahr.) of its temperature. However, some individuals may be cooled without being exhausted by convulsions. I have experimented on a great many which were in this condition. For more than eight years, without intending to make experiments on this subject, I have had the opportunity of stating, on more than a hundred adult rabbits or guinea-pigs, used for other researches, that, when their temperature is diminished, the duration of their life, when they are completely deprived of breathing, is decidedly longer than in the normal state. Whatever has been the cause of the cooling, the effect has been the same. In these numerous experiments, the animal heat has been diminished, either by a disease, by a poison, by an injury in the nervous centres, by the immersion of the animal in melted ice, or by the application of a layer of oil, essence, or gelatine, to the whole skin of the animal.

The best mode of cooling a non-hybernating warm-blooded animal is to make the following experiment, in a room where the temperature of the air is not far from freezing point. I remove the superior part of the cranium of a mammal or of a bird, and afterwards cut the brain, slice by slice, from the anterior to the posterior extremity. After the ablation of the cerebrum and the cerebellum, the animal is put on the floor, where it is left perfectly quiet for an hour or two. If the experiment is made on a rabbit or a guinea-pig, the temperature of the animal then falls from 40° Cent. (104° Fahr.) to 30° or 35° Cent. (86 or 95° F.) A ligature being put around the trachea, I find that the animal is then able to live generally from six to 8 or 12 minutes.

The more the animal heat is diminished, the more, in general, is the resistance to asphyxia, except in cases where cooling has been produced too quickly.

On four adult rabbits, I put a ligature around the trachea, and have obtained the following results:

TABLE XI.

| Nos. | Temperature of the animals. | Duration of life. |
|---|---|---|
| 1, | 39½° Cents., (103° Fahr.) | 3½′ |
| 2, | 35 " 95 " | 6 |
| 3, | 30½ (between 86 and 87 Fahr.) | 10 |
| 4, | 25 Cents., (77 Fahr.) | 14 |

On three guinea-pigs I put a ligature around the trachea, and found the following facts:

TABLE XII.

| Nos. | Temperature of the animals. | Duration of life. |
|---|---|---|
| 1, | 40° Cents., (104° Fahr.) | 2¾′ |
| 2, | 35 " 95 " | 5½ |
| 3, | 30 " 86 " | 12 |

The longest persistence of life, after the cessation of breathing which I have found in adult non-hybernating animals, has been in a cat aged five months, and whose temperature had been diminished to 19° Cent. (about 67° Fahr.) in consequence of the laying bare of the abdominal viscera, and of the ablation of the cerebrum by small parts.

I will say only a few words about my experiments on adult birds.

Two pigeons were dipped into melting ice. In one of them, whose temperature had fallen from 42° Cent. (108° Fahr.) to 35° Cent. (95° Fahr.) life lasted six minutes after the ligature of the trachea. In the other, life lasted nine minutes; its temperature had fallen from 42° Cent. (108° Fahr.) to 30° Cent. (86° Fahr.)

I have obtained nearly the same results in experimenting on fowls and on ducks.

Physicians are generally astonished at seeing that men attacked with cholera are able to live almost without breathing. My experiments show that the principal, if not the only cause of the power living with so deficient a respiration, is the diminution of temperature. The same thing occurs in the last hour of life in many cases of diseases, and particularly those of the brain, of the respiratory organs, and in that dreadful disease of children called *scleroma.*

It is easy to understand how a considerable breathing becomes less and less necessary when the temperature of the animals under experiment is diminishing.

Whatever may be the function of oxygen, it is positive that most of the chemical changes which take place in the living animals are accompanied with a consumption of oxygen. If so, when these chemical changes are diminished, the consumption of oxygen ought to diminish. Now, as every diminution of the temperature of an animal produces a proportionate diminution in nearly all the acts of organic and animal life, and as these acts are all accompanied by chemical changes in which oxygen is consumed, it follows that, when they diminish, the consumption of oxygen also diminishes.

In the act of running, or in a rapid walk, we consume much more oxygen than in a state of rest. What we call rest is merely a state of diminished activity; and when the temperature of a mammal has been cooled down, the activity of most of the functions is much more diminished than in the most complete rest. It results from this fact, that when a warm blooded animal has lost a notable part of its ordinary warmth, its consumption of oxygen is much less than in a state of rest.

If the ligature of the trachea is made simultaneously on two adult mammals of the same species, one of them being at its normal temperature, and the other at a temperature of 5° Cent. (9° Fahr.) lower, we observe that this last one lives six, seven or eight minutes, and the other from one and a half to three minutes. If we suppose the quantity of oxygen existing in the lungs and in the blood of these two animals is precisely alike in both ; and if we admit that death occurs in asphyxia, either from want of oxygen or by an action of carbonic acid, in both cases these two animals ought to live a different length of time, because the consumption of oxygen contained in blood and the production of carbonic acid are quicker in one of them than in the other. This explanation is so true, that when movements are excited in the cooled animal, after the beginning of asphyxia, it dies sooner than when it remains in rest.

From the facts and reasonings which are related in this note I draw the following conclusions :

1st. The temperature of newly-born warm-blooded animals has a great influence on the duration of their life when they are asphyxiated.

2d. There are very considerable differences in the duration of life in asphyxiated animals of different species, even when the experiment is performed in the same medium, and while their temperature is at the same degree.

3. The degree of the temperature of adult warm-blooded animals has also a great influence on the duration of their life when they are asphyxiated.

4th. The influence of the animation of the temperature of a warm-blooded animal on the duration of its life in asphyxia, explains the persistence of life in man, in cases of cholera, scleroma, and of some other diseases, when respiration is much diminished.

XVIII.—ON THE CENTRAL SEAT OF GENERAL AND OF TACTILE SENSIBIBITY, AND ON THE VALUE OF CRIES AS MANIFESTATIONS OF PAIN.*

To determine where is the seat of perception and of volition is one of the greatest physiological questions. Flourens maintains

* See Comptes Rendus de l'Acad. des Sciences, 1849. t. xxix. p. 672.

that this seat is in the central lobes. Many physiologists, among whom are Bouillaud, Gerdy and Longet, have published papers against the doctrine of Flourens. Their only important argument is that mammals deprived of their whole encephalon, except the medulla oblongata and the pons varolii, continue to possess the faculty of perceiving sensations, and that the perception of pain is then manifested by cries and agitation. When the encephalon, says Longet,* is so much mutilated, in rabbits and dogs, that only the pons varolii and the medulla oblongata remain, in the cranial cavity, these animals, although they seem to be in a deep coma, are still able to agitate themselves, and to cry plaintively, under the influence of strong external irritations; but if a sufficiently deep alteration is made in the pons varolii there is an immediate cessation of the cries and of the agitation; it merely remains an animal in whom the circulation, the respiration and the other nutritive functions are momentarily accomplished.

On cats, rabbits and guinea-pigs, I have obtained a completely different result in performing that experiment. After I had removed, slice after slice, and from forwards backwards, the whole encephalon, except only the medulla oblongata, I have found that the mutilated animal, not only is much agitated, but cries plaintively when it is pinched. If the medulla oblongata is also removed, the cries cease, but the agitation still continues.

According to these experiments, it is evident that the pons varolii is not the only *seat of sensibility*, *the centre of perception of tactile impressions*, as Longet calls it, and that either the medulla oblongata is the seat of general sensibility or the cries do not prove that there is a perception.

Longet considers that the pons varolii is not only the centre of perception for sensation of pain, but also for tactile impressions. As to the tactile sensations he does not give the slightest appearance of proof. Certainly neither the cries nor the agitation are sufficient to authorize the opinion that the animal has felt a sensation of tact. If the existence of cries could prove that there is a perception of a tactile sensation, I should have to conclude from my experiments that the medulla oblongata is the seat of

* Traite de Physiol. Paris. 1850. t. ii, B. p. 38.

the faculty of perception of tactile sensations, for there are cries after the removal of the whole encephalon except the medulla oblongata, and there are no more after the removal of this last organ. If, instead of drawing conclusions from the existence of cries, we take notice only of the agitation, we are bound to conclude that the spinal cord is the seat of the faculty of perception of tactile sensations, for after this organ has been separated from the medulla oblongata, agitation takes place when a limb is pinched.*

Cries and agitation may be attributed to a property of the nervous centres, which is completely different from the faculty of perception of painful or tactile sensations. That property is the reflex faculty of the true spinal marrow;† it is the property of uniting, in co-ordinate movements isolated muscular contractions,‡ which is called by German physiologists the faculty of adaptation to an end. That property manifests itself by movements similar to those executed by unmutilated animals when they feel a pain; and it happens, sometimes, that these reflex movements are less disordered than the movements consecutive to a violent pain in an unmutilated animal. The *agitation* of the animals deprived of all the parts of their encephalon is merely the result of an action of the reflex faculty.

The cries also appear to exist only in consequence of a reflex action. This appears to be difficult to be proved, and it will seem nearly impossible to admit that cries may be produced by an animal that has felt no pain, or that has not had the will of crying. We may consider a cry as a noise produced in the larynx, as many times a quick expiration is performed when the vocal cords are stretched. Now as the tension of these cords and the expiration is produced by muscular contractions, it is easy to understand that these contractions are produced by a reflex action as well as the contractions of the muscles of the limbs.

For those who know that hiccup, coughing, sneezing, vomiting

* This conclusion is maintained as true by Sénac, Caldani, Kay, Legallois, Paton, J. W. Arnold and many others.

† As Dr. Marshall Hall calls it.

‡ That is the name given by Flourens more than 25 years ago.

and so forth, frequently are mere reflex phenomena, there ought to be no difficulty in admitting that crying is a pure reflex action.

If we here use the expressive language of Flourens, we will say, that the medulla oblongata has the faculty of uniting in a co-ordinate movement, the contractions of the expiratory muscles and that of the tensor muscles of the glottis.

From the facts and reasonings contained in this note I will now draw the following conclusions:

1st, That the experiment by which many physiologists have endeavored to prove that the cerebral lobes are not the exclusive seat of the perceptions, do not give such a proof.

2d, That animals can cry after the removal of the whole encephalon, except only the medulla oblongata.

3d, That the existence of cries cannot prove that there is a perception of pain, because cries result from muscular contractions which may be pure reflex actions.

4th, That there is no proof that the pons varolii is the centre of perceptions either of touch or of pain.

5th, That if it is admitted that cries prove that there is a perception of pain, we should have to admit that the medulla oblongata is also a centre for these perceptions.

(To be continued.)

